# Influence of maxillofacial skeletal morphology on difficult laryngoscopy

**DOI:** 10.1186/s12871-025-02997-0

**Published:** 2025-04-01

**Authors:** Reiko Sekino, Minami Hikida, Keiji Shinozuka, Maki Nagasaki, Akemi Kusano, Morio Tonogi, Shunichi Oka

**Affiliations:** 1 Department of Anesthesiology, Nihon University School of Dentistry, 1-8-13, Kanda- Surugadai, Chiyoda-ku, Tokyo, 101-8310 Japan; 2 First Department of Oral and Maxillofacial Surgery, Nihon University School of Dentistry, 1-8-13, Kanda-Surugadai, Chiyoda-ku, Tokyo, 101-8310 Japan

**Keywords:** Difficult laryngoscopy, Maxillofacial skeletal morphology, Sassouni classification, orthognathic surgery

## Abstract

**Background:**

Although mandibular retrognathia has been recognized as one of the predictors of difficult laryngoscopy, its definition remains vague, with no clearly established skeletal evaluation systems. The Sassouni classification system, used to categorize the maxillofacial morphology systematically (nine types), can be easily performed using preoperative radiographic findings. This study aimed to investigate the relationship between difficult laryngoscopy and Sassouni type 5, a group characterized by a small mandible and a large overbite.

**Methods:**

This retrospective study comprised patients diagnosed with jaw deformities who underwent orthognathic surgery for malocclusion under general anesthesia at our clinic. The patients were divided into two groups: Sassouni 5 and non-Sassouni 5 (types 1, 2, 3, 4, 6, 7, 8, and 9). Difficult laryngoscopy was evaluated by examining the degree of difficulty in laryngeal exposure, which was defined as grades 3 and 4 based on the Cormack-Lehane (CL) classification. Additionally, we evaluated the relationships between the Sassouni 5 group and three predictors of difficult laryngoscopy (Mallampati classification, Wilson score, and hyomental distance [HMD]).

**Results:**

Of the 187 patients included in this study, 44 belonged to the Sassouni 5 group, and the remaining 143 belonged to the non-Sassouni 5 group. The proportion of patients with CL grade 3 or higher was significantly higher in the Sassouni 5 group (*n* = 9; 20.5%) than in the non-Sassouni 5 group (*n* = 6; 4.2%). Furthermore, 10 (22.7%) patients in the Sassouni 5 group had a Mallampati score of 3 or higher, 44 (100%) had a Wilson score of 2 or higher, and 38 (96.7%) had an HMD of less than 3 fingerbreadths. The corresponding numbers in the non-Sassouni 5 group were 8 (5.6%), 48 (33.6%), and 43 (30.1%), respectively. The incidence of difficult laryngoscopy in the Sassouni 5 group was significantly higher than that in the non-Sassouni 5 group (*p* < 0.001).

**Conclusions:**

These findings indicate that the incidence of difficult laryngoscopy can be predicted using the Sassouni classification, which can be easily analyzed using lateral cephalograms obtained routinely before corrective surgical procedures. The Sassouni 5 group could be used as an important predictive tool in clinical practice.

**Supplementary Information:**

The online version contains supplementary material available at 10.1186/s12871-025-02997-0.

## Introduction

According to the American Society of Anesthesiologists (ASA) guidelines, difficult airway is defined as “a clinical situation in which a trained anesthesiologist encounters difficulty with mask ventilation, tracheal intubation, or both,” and is classified into four categories: difficult mask ventilation, difficult laryngoscopy, difficult tracheal intubation, and failed intubation [1]. Orthognathic surgery is typically performed on healthy young individuals; however, it often presents challenges related to difficult laryngoscopy due to skeletal issues. According to the ASA Task Force, difficult laryngoscopy is defined as a Cormack-Lehane (CL) classification of grade 3 and 4 [2]. Although mandibular retrognathia has been recognized as one of the predictors of difficult laryngoscopy, its definition remains vague, and no clear skeletal evaluation has been established so far [3, 4]. Patients with jaw deformities undergo lateral cephalometric radiography to determine the surgical approach for orthognathic surgery; simultaneously, the skeletal morphology is assessed preoperatively and categorized into nine types using the Sassouni classification system [5, 6]. Demonstrating a relationship between this classification system and difficult laryngoscopy can aid in predicting the preoperative outcomes using only lateral cephalometric images, thereby simplifying and expediting the assessment process before meeting the patient.

This study aimed to investigate the relationship between difficult laryngoscopy and Sassouni type 5, a group characterized by a small mandible and a large overbite.

## Materials and methods

### Study design and setting

This retrospective study was approved by the Ethics Committee of Nihon University School of Dentistry (approval number: EP21D013) and conducted in accordance with the principles of the Declaration of Helsinki. The dental university hospital serves as a collaborative hub for the Departments of Oral and Maxillofacial Surgery, Dental Anesthesiology, and Orthodontics, who work together to treat patients with jaw deformities. General anesthesia is managed by dental anesthesiologists for all patients. Skeletal evaluations of patients with jaw deformity are conducted by orthodontists using the Sassouni classification during the initial consultation.

### Participants

Patients diagnosed with jaw deformities at the Department of Oral and Maxillofacial Surgery between January 2018 and December 2021 who underwent orthognathic surgery under general anesthesia for malocclusion were included in this study. The inclusion criteria were as follows: underwent treatment under general anesthesia, performed intubation and extubation in the operating room, and underwent orthognathic surgery. Patients with congenital skeletal abnormalities not classified by the Sassouni system or those with significant facial asymmetry were excluded. Furthermore, individuals with a body mass index (BMI) exceeding 25 kg/m² were not included in order to focus on the correlation between skeletal assessment and difficult laryngoscopy.

### The Sassouni analysis

In the Sassouni analysis, the midpoint of the region where the basicranial (parallel to the supraorbital plane), palatal, occlusal, and mandibular planes converged was defined as the O point. An arc with the O-nasion as the radius was drawn and used to evaluate malpositioning in the maxillofacial region. In a harmonious profile, the anterior nasal spine (ANS), incisor (IS), and pogonion (Pog) lines align along an arc. The horizontal classification utilized this arc and categorized maxillofacial surfaces with the ANS, IS, and Pog on the arc line as skeletal class I. Skeletal class II was defined by the presence of maxillary prognathism, mandibular retraction, or a combination of both relative to the arc line. Skeletal class III involved maxillary retraction, mandibular prognathism, or a combination of both. Vertical classification relied on the overbite level: 0–4 mm, average bite; >4 mm, deep bite; and < 0 mm, open bite.

**Fig. 1:**
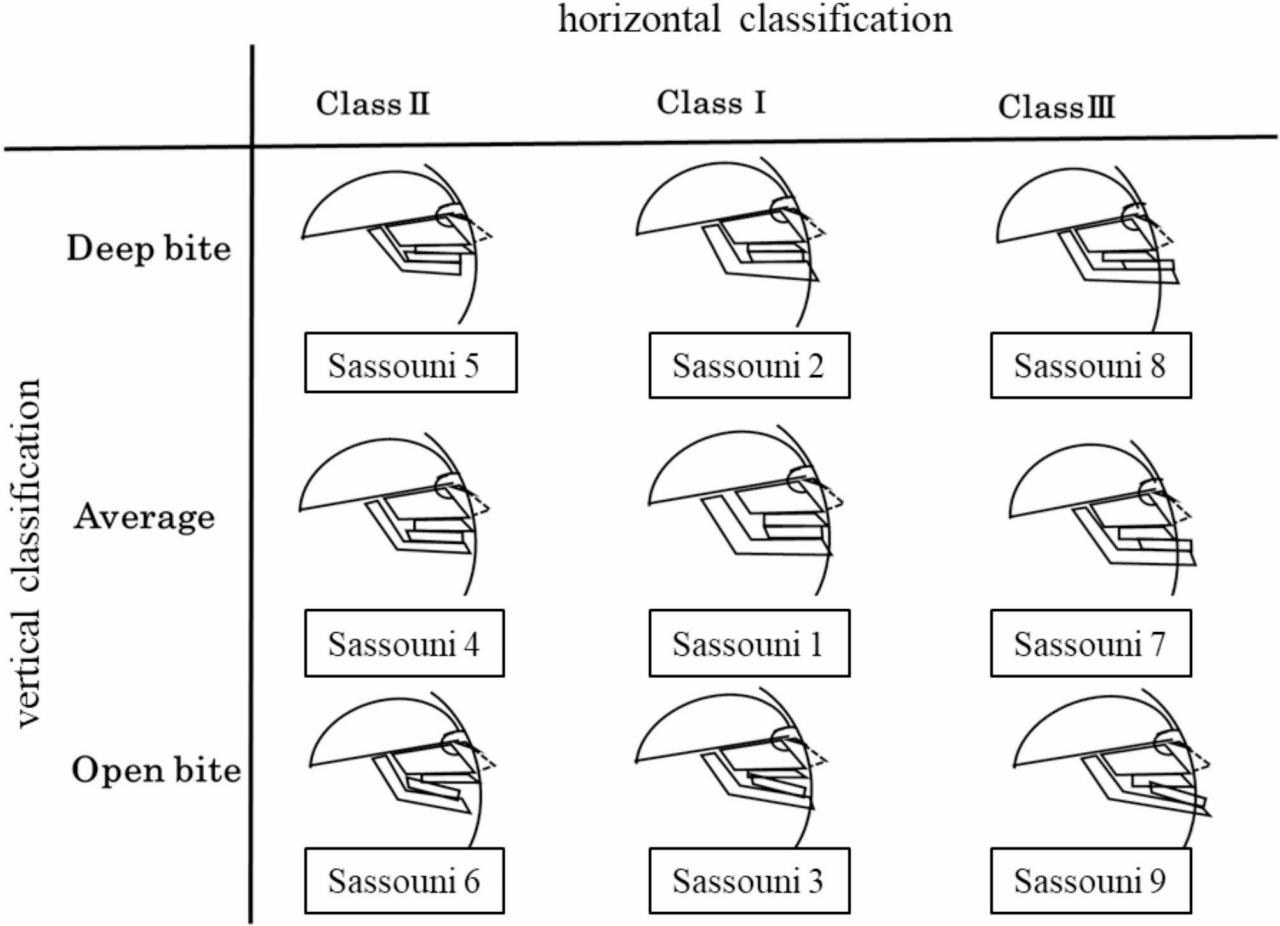
The Sassouni classification

By combining these horizontal and vertical classifications, the maxillofacial region was systematically classified into nine groups (Fig. 1) as follows: Sassouni 1, skeletal class I and average bite; Sassouni 2, class I with a deep bite; Sassouni 3, class I with an open bite; Sassouni 4, class II with an average bite; Sassouni 5, class II with a deep bite; Sassouni 6, class II with an open bite; Sassouni 7, class III with an average bite; Sassouni 8, class III with a deep bite; and Sassouni 9, class III with an open bite [5, 6].

Difficult laryngoscopy was evaluated by examining the degree of difficulty in laryngeal exposure, which was defined as grades 3 and 4 based on the CL classification. In the current study, the number of patients with CL grade 3 or 4 in the Sassouni 5 group was significantly higher than in the remaining groups (Supplementary Figure S1). Therefore, we focused on evaluating the relationship between each evaluation criterion for predicting difficult laryngoscopy and the Sassouni 5 group. Consequently, the patients were divided into two main groups: the Sassouni 5 group and the non-Sassouni 5 group (which included patients with the remaining eight Sassouni types).

### Variables and potential risk factors

The ASA Practice Guidelines for the Management of a Difficult Airway states that if a trained provider requires more than three attempts at intubation or if intubation attempts last longer than 10 min, it is considered a difficult intubation [1]. However, since all intubations in this study were performed via the nasal route, factors such as nasal morphology, including nasal septum deviation or nasal polyps, as well as complications like epistaxis, could influence the assessment of a difficult intubation. Therefore, this study focused on evaluating the difficulty of laryngeal exposure, defined as CL grades 3 and 4 based on the ASA Task Force.

Data on age, sex, BMI, jaw mobility, spine mobility, CL classification, and preoperative predictors of difficult airways were collected from the patients’ medical records by one of the investigators of this study (R.S).Regarding age, it is known that both pediatric and elderly patients are at increased risk for difficult laryngoscopy [7, 8]. However, due to the eligibility criteria for orthognathic surgery based on age, no patients younger than 15 or older than 65 were included in this study.

Jaw and spine mobility were considered as potential factors influencing the outcomes. Temporomandibular joint evaluation and treatment are routinely performed during the initial consultation for orthognathic surgery planning. Jaw mobility was assessed based on the “3-3-2 rule” [9], specifically evaluating whether the patient could achieve a mouth opening of 3 fingerbreadths or more. Spine mobility was assessed by determining whether patients could flex their neck to at least 90° [10]. Patients with cervical spine disorders are not eligible for orthognathic surgery at our hospital.

The preoperative predictors of difficult laryngoscopy included the Mallampati classification [11], Wilson score [10], and hyomental distance (HMD) [12]. The Mallampati classification, used to predict difficult airways by observing the oropharyngeal structures, classifies patients into four categories: Class I, II, III, and IV. Generally, Class III or higher is considered indicative of a risk for difficult laryngoscopy.

The Wilson score is an assessment method used to predict the difficulty of laryngoscopy. It evaluates five factors: neck movement, mandibular retrusion, mouth opening, mandibular protrusion, and body weight. Each factor is scored from 0 to 2, and the total score correlates with the risk of difficult laryngoscopy. It is the most widely studied composite score, and a value of ≥ 2 indicates a difficult laryngoscopy.

The HMD is the distance between the hyoid bone and mentum and can be measured with the head in the neutral position. The HMD assesses the risk of difficult laryngoscopy when the measurement is less than 3 fingerbreadths (< 3 to < 5.5 cm). As the measurement influenced by the height and size of the body, it is performed using the patient’s fingers.

### Statistical analysis

Comparison analysis was performed using the independent t-test for continuous variables and Chi-square or Fisher exact test for non-continuous variables. Statistical analyses were performed using SPSS software (IBM Corp., Armonk, NY, USA), and the level of significance was set at *p* < 0.05.

## Results

### Baseline findings

**Fig. 2:**
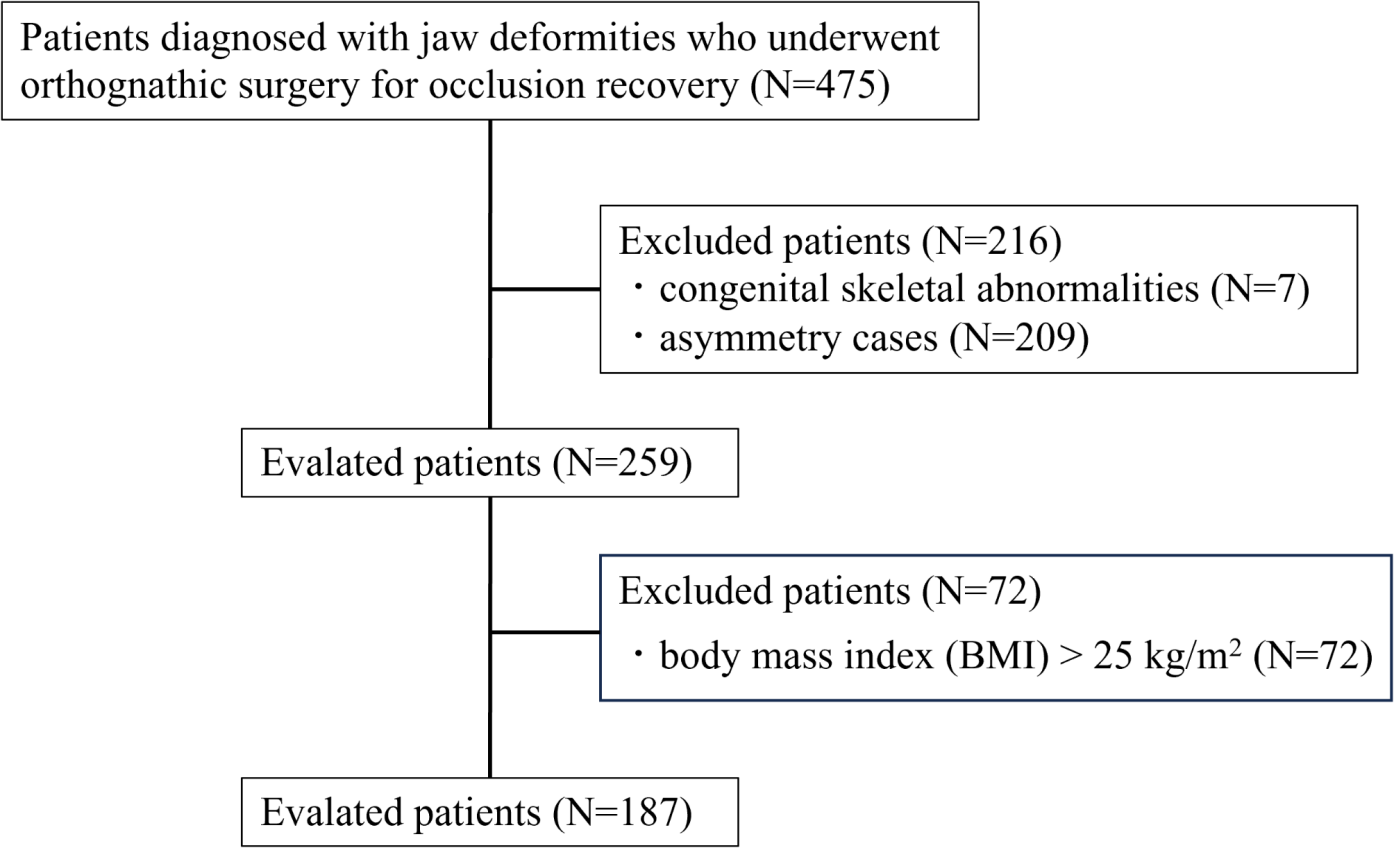
Flow chart of the patient selection process

A total of 475 patients diagnosed with jaw deformities who underwent orthognathic surgery for occlusal restoration at the Department of Oral and Maxillofacial Surgery were included in this study. Among them, 7 with congenital skeletal abnormalities, 209 with significant facial asymmetry, and 72 with a BMI of ≥ 25 kg/m² were excluded. Finally, 187 patients (44 in the Sassouni 5 group and the remaining 143 in the non-Sassouni 5 group), were included in this study (Fig. 2).

Twelve patients (27.4%) in the Sassouni 5 group and 42 patients (29.4%) in the non-Sassouni 5 group were males, with no significant difference in gender distribution between the two groups. Similarly, no significant differences in age or BMI were observed between the groups.

All patients in this study demonstrated a mouth opening of at least 3 fingerbreadths. Furthermore, all patients achieved a neck flexion of 90° or more (Table 1).

**Table 1:** Characteristics of the patients at enrollment

	Total	Sassouni 5	Non-Sassouni 5	*p*-value
	*N* = 187	*n* = 44	*n* = 143	
Males, n (%)	54 (28.9%)	12 (27.2%)	42 (29.4%)	0.689 *
Mean age ± SD	27.7 ± 8.14	29.6 ± 7.41	27.3 ± 8.24	0.156
Mean BMI (kg m^− 2^)	20.7 ± 2.15	20.5 ± 2.38	20.8 ± 2.11	0.493
Jaw mobility				
*able*	187	44	143	
*unable*	0	0	0	
Spine mobility				
*>90°*	187	44	143	
*≤90°*	0	0	0	

N, total number of patients; n, number of patients in the groups; SD, standard deviation; BMI, body mass index. Jaw mobility: an incisor distance of 3 fingerbreadths. Data were tested by T-test and *chi square test.

### Association between difficult laryngoscopy and the Sassouni 5 group

The incidence of difficult laryngoscopy, indicated by the number of patients with CL grade 3 or higher, was significantly higher in the Sassouni 5 group (9/44; 20.5%) than in the non-Sassouni 5 group (6/143; 4.2%; Table 2).

**Table 2:** Associations between difficult laryngoscopy and the Sassouni 5 and non-Sassouni 5 groups based on the Cormack–Lehane grading scale

	Total	Sassouni 5	Non-Sassouni 5	*p*-value
	*N* = 187	*n* = 44	*n* = 143	
CL grade, n (%)				
< 3	172 (92.0%)	35 (79.5%)	137 (95.8%)	
≥3	15 (8.0%)	9 (20.5%)	6 (4.2%)	< 0.001

N, total number of patients; n, number of patients in the groups; CL grade, Cormack–Lehane grading scale. Difficult laryngoscopy was defined as CL grade ≥ 3. Data were tested by Fisher exact test

Additionally, among patients predicted to have difficult laryngoscopy based on known predictors in the Sassouni 5 group, 10 (22.7%) had a Mallampati score of 3 or higher, 44 (100%) had a Wilson score of 2 or higher, and 38 (96.7%) had an HMD of less than 3 fingerbreadths (Table 3). The corresponding numbers in the non-Sassouni 5 group were 8 (5.6%), 48 (33.6%), and 43 (30.1%), respectively.

**Table 3:** Associations between the predictors of difficult laryngoscopy and the two Sassouni groups

Predictors	Total	Sassouni 5	non-Sassouni 5	*p*-value
	*N* = 187	*n* = 44	*n* = 143	
Mallampati Score				
< 3	169 (90.4%)	34 (77.3%)	135 (94.4%)	
≥3	18 (9.63%)	10 (22.7%)	8 (5.59%)	< 0.001
Wilson score				
< 2	95 (50.8%)	0	95 (66.4%)	
≥2	92 (49.2%)	44 (100%)	48 (33.6%)	< 0.001
HMD				
<3 fingerbreadths	81 (43.3%)	38 (96.7%)	43 (30.1%)	
≥3 fingerbreadths	106 (56.7%)	6 (3.3%)	100 (69.9%)	< 0.001

N, total number of patients; n, number of patients in the groups; HMD, hyomental distance. Data were tested by Fisher exact test.

### Sensitivity of Sassouni 5 and other predictors of difficult laryngoscopy

The sensitivity of the Sassouni 5 group for predicting difficult laryngoscopy was 60.0%, while the specificity was 79.7% (Table 4). Likewise, the sensitivity a Mallampati score of ≥ 3 was 26.7%, a Wilson score of ≥ 2 was 73.3%, and an HMD of < 45 mm was 73.3%; the corresponding specificity values were 92.9%, 52.9%, and 59.3%, respectively (Table 4).

**Table 4:** The sensitivity of the Sassouni 5 group and other predictors of difficult laryngoscopy

	Sensitivity	Specificity	PPV	NPV
**Sassouni 5**	60.0%	79.7%	20.5%	95.8%
** Mallampati Score ≥ 3**	26.7%	92.9%	22.2%	93.4%
** Wilson Score ≥ 2**	73.3%	52.9%	12.0%	95.8%
**HMD < 3 finger breadths**	73.3%	59.3%	13.6%	96.2%

PPV, positive predictive value; NPV, negative predictive value; HMD, hyomental distance. Difficult laryngoscopy was defined as CL grade ≥ 3.

## Discussion

Mandible retrognathia is one of the predictors of difficult laryngoscopy; however, its definition remains ambiguous, and evaluations based on skeletal morphology are lacking. This study aimed to establish a preoperative risk assessment indicator for difficult laryngoscopy using cephalometric images (Sassouni analysis) routinely taken during preoperative orthodontic evaluation for patients with jaw deformities. Relationships between the nine Sassouni groups and CL classification were examined. The proportion of patients with CL grade 3 or higher was significantly higher in the Sassouni 5 group than in the non-Sassouni 5 group. The sensitivity and specificity of the Sassouni 5 group to difficult laryngoscopy were 60.0% and 79.7%, respectively, with a positive predictive value of 20.5%, second only to the Mallampati classification. These findings suggest that patients classified as Sassouni type 5 based on skeletal morphology are at higher risk of difficult laryngeal exposure.

Mandibular retrognathia has been reported as a factor associated with difficult laryngoscopy [3, 4, 10]. However, the diagnostic criteria for mandibular retrognathia are often broad, as seen in the Wilson score, and rely heavily on the subjective judgment of the evaluator, lacking clear and standardized criteria. While several skeletal morphologies fall under the category of mandibular retrognathia, none have been thoroughly investigated. The Sassouni classification categorizes skeletal morphology with precise criteria. Vertically, it defines normal overbite as strictly between 0 and 4 mm. Horizontally, maxillary overjet or mandibular retrognathia is classified based on whether the Pog lies inside the arc, defined by a radius extending from the nasion to the midpoint of four planes: the cranial base, palatal plane, occlusal plane, and mandibular plane [5, 6]. In the current study, the number of patients with CL grade 3 or 4 in the Sassouni 5 group was significantly higher than those in the other Sassouni groups (Supplementary Figure S1). Based on this finding, we conducted a retrospective study focusing on Sassouni 5. Generally, the proportion of cases with CL 3 or higher ranges between 1.5% and 8.5% [3, 13]; however, in the current study, the Sassouni 5 group demonstrated a significantly higher proportion at 20.5%. In contrast, the proportion in the non-Sassouni 5 group was 4.2%, which aligns closely with the general reported range.

The Mallampati score is widely recognized as a useful tool for predicting difficult laryngoscopy, with a higher prevalence of CL grade 3 or higher in patients with a Mallampati score of III or higher [14, 15]. Furthermore, numerous studies have reported on the sensitivity and specificity of this method for predicting difficult laryngoscopy, and reported a sensitivity of 35.0–66.7% for a Mallampati score of III or higher and a specificity of 78.8–91.0% [16–18]. However, in the present study, the sensitivity of this score for predicting a CL grade of ≥ 3 was lower than previously reported. This discrepancy may be attributed to the impact of skeletal morphology deviations, characteristic of jaw deformity patients, on the surrounding soft tissues.

The HMD has been reported as a reliable indicator with no significant differences based on age or sex and is often used with other predictive tools, such as the 3-3-2 rule, to predict difficult laryngoscopy [19, 20]. Reports suggest that the sensitivity of HMD alone is 40%, with a specificity of 78% for difficult laryngoscopy [21]. Similarly, the Wilson score is reported to have a sensitivity of 36% and a specificity of 98.5% for difficult laryngoscopy [22]. In the current study, we calculated the sensitivity and specificity of these predictors with regard to CL grade 3 or 4. The sensitivity values for both HMD and the Wilson score were higher than those reported previously. Both indicators include parameters evaluating mandibular retrognathia, and considering that Sassouni 5 also demonstrated high sensitivity, it is suggested that a CL grade of 3 or higher is closely related to mandibular retrognathia.

The proportion of cases with an HMD of < 3 fingerbreadths or a Wilson score of ≥ 2 was higher than those with an HMD of ≥ 3 fingerbreadths or a Wilson score of < 2. Thus, the positive predictive values of HMD and the Wilson score were lower than those of the Mallampati classification or Sassouni 5. These findings indicate that Sassouni 5 can potentially serve as a preoperative risk assessment tool for predicting difficult laryngoscopy.

In recent years, the evaluation of difficult laryngoscopy using X-ray imaging has involved methods with three-dimensional CT digital quantitative measurements of anatomical structures, such as the mandible, tongue, and airway [23]. Radiological evaluation using lateral cephalometric images and CT has shown that factors such as the tongue area measured on the osteomeatal unit CT, the distance from the mandibular alveolar line to the hyoid bone, and the distance from the inner margin of the mandible to the hyoid bone on lateral cephalometric images are associated with difficult laryngoscopy [24]. However, these evaluation methods are complex and time-consuming. Although radiological assessments like cephalometry provide only two-dimensional evaluations, cephalometric imaging, and analysis using the Sassouni classification are routinely performed during preoperative orthodontic planning for patients undergoing orthognathic surgery. Incorporating the Sassouni classification into preoperative assessments for difficult laryngoscopy will enable simpler and earlier prediction using only cephalometric images, even before meeting the patient. This approach facilitates timely preparation and appropriate interventions, thereby improving clinical efficiency and safety.

The present study has several limitations. The presence of macroglossia represents an uncontrolled confounding factor. Congenital conditions such as the Beckwith-Wiedemann syndrome, which presents with macroglossia, were excluded from the study; nonetheless, the absence of clear diagnostic criteria for macroglossia indicates that this factor was not controlled in the included cases. Additionally, the observational study design and the small sample size of the Sassouni 5 group present limitations. Moreover, the large disparity in the number of patients between the two groups might have affected the results. The inability to adequately control confounding variables and consider potential interactions between variables may result in spurious associations or inaccuracies in conclusions about the relationship between exposure and outcome. These factors could compromise the validity of the study’s findings.

## Conclusion

In this study, we predicted the incidence of difficult laryngoscopy using the Sassouni classification, which can be easily analyzed using lateral cephalograms obtained routinely before corrective surgical procedures. In particular, patients in the Sassouni 5 group exhibited significantly difficult laryngoscopy. Thus, incorporating the Sassouni analysis in conjunction with established predictors of difficult laryngoscopy can enhance the reliability of predicting the condition, thereby facilitating the preparation of alternative instruments to laryngoscopes, enabling strategic planning for conscious laryngoscopy, and mitigating potential serious complications during anesthesia management.

## Electronic supplementary material

Below is the link to the electronic supplementary material.

Supplementary Material 1

## Data Availability

The data that support the findings of this study are available from the corresponding author upon reasonable request.

## Abbreviations

ANS: Anterior nasal spine
BMI: Body mass index
CL: Cormack–Lehane
HMD: Hyomental distance
IS: Incisor
Pog: pogonion
