# Supplementary material for: Influence of maxillofacial skeletal morphology on difficult laryngoscopy
Source: BMC Anesthesiol. 2025 Apr 1;25:147. doi: 10.1186/s12871-025-02997-0 (PMC11963286; doi:10.1186/s12871-025-02997-0)
Supplement: Supplementary file 1 — Supplementary Material 1 [file 12871_2025_2997_MOESM1_ESM.docx]

Supplemental Figure 1.

**
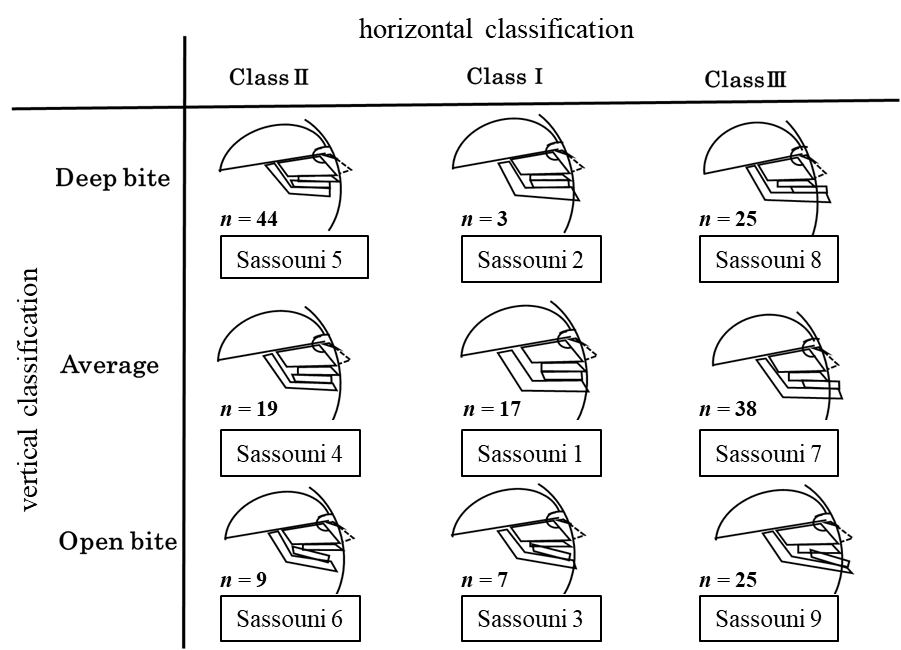
A**

**B　
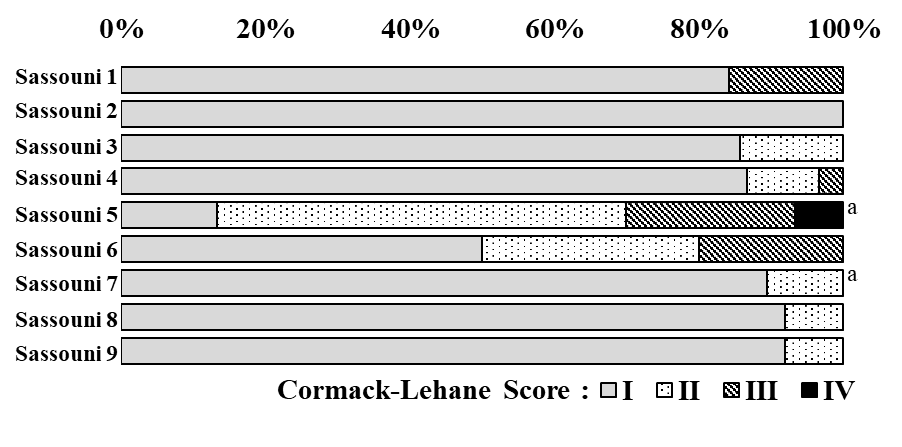
**

The Sassouni classification. (A). Description of the Sassouni classification system and the number of patients in each group. (B). Comparison of the Cormack–Lehane scores among the nine groups using Fisher's exact test with Bonferroni correction. ^a^Significant differences (*P* < 0.05) in the number of patients with CL grade 3 or 4 were observed between the Sassouni 5 group and the other Sassouni groups, indicating Sassouni 5 as a potential risk indicator for difficult laryngoscopy.
